# Recurrence risk prediction of acute coronary syndrome per patient as a personalized ACS recurrence risk: a retrospective study

**DOI:** 10.7717/peerj.14348

**Published:** 2022-11-15

**Authors:** Vungsovanreach Kong, Oui Somakhamixay, Wan-Sup Cho, Gilwon Kang, Heesun Won, HyungChul Rah, Heui Je Bang

**Affiliations:** 1 Department of Big Data, Chungbuk National University, Cheongju, South Korea; 2 Department of Management Information Systems, Chungbuk National University, Cheongju, South Korea; 3 Department of Health Informatics and Management, College of Medicine, Chungbuk National University, Cheongju, South Korea; 4 Cybrebain Research Section, Electronics and Telecommunications Research Institute, Daejeon, South Korea; 5 Research Institute of Veterinary Medicine, Chungbuk National University, Cheongju, South Korea; 6 Department of Rehabilitation Medicine, College of Medicine, Chungbuk National University, Cheongju, South Korea

**Keywords:** Acute coronary syndromes, Logistic regression, Personalized recurrence risk

## Abstract

Acute coronary syndrome (ACS) has been one of the most important issues in global public health. The high recurrence risk of patients with coronary heart disease (CHD) has led to the importance of post-discharge care and secondary prevention of CHD. Previous studies provided binary results of ACS recurrence risk; however, studies providing the recurrence risk of an individual patient are rare. In this study, we conducted a model which provides the recurrence risk probability for each patient, along with the binary result, with two datasets from the Korea Health Insurance Review and Assessment Service and Chungbuk National University Hospital. The total data of 6,535 patients who had been diagnosed with ACS were used to build a machine learning model by using logistic regression. Data including age, gender, procedure codes, procedure reason, prescription drug codes, and condition codes were used as the model predictors. The model performance showed 0.893, 0.894, 0.851, 0.869, and 0.921 for accuracy, precision, recall, F1-score, and AUC, respectively. Our model provides the ACS recurrence probability of each patient as a personalized ACS recurrence risk, which may help motivate the patient to reduce their own ACS recurrence risk. The model also shows that acute transmural myocardial infarction of an unspecified site, and other sites and acute transmural myocardial infarction of an unspecified site contributed most significantly to ACS recurrence with an odds ratio of 97.908 as a procedure reason code and with an odds ratio of 58.215 as a condition code, respectively.

## Introduction

Acute coronary syndromes (ACS) refer to a range of conditions in which atherosclerotic plaque builds up by fatty substances inside a patient’s coronary arteries, which reduces or restricts the blood flow (and therefore the delivery of oxygen) to the heart (Overbaugh, 2009). Insufficient blood flow to the heart may lead to heart attack, the most common condition of ACS, which will destroy part of the patient’s cardiac muscle. ACS has become a challenge and concern in the health sector all over the globe. In the United States, more than 121 million adults, which is equal to 48% of total adults, suffered from heart disease in 2016 (Benjamin et al., 2019). According to data from the Centers for Disease Control and Prevention (CDC), 1.5 million people of the United States population suffer from heart attacks and strokes each year, which contributes to more than $320 billion in annual healthcare costs and lost productivity. By 2030, this cost is projected to rise to $818 billion, while lost productivity costs will rise to $275 billion (Giedrimiene & King, 2017). The high recurrence risk of patients with known coronary heart disease (CHD) has led to a focus on the importance of post-discharge care and secondary prevention of CHD (Yudi et al., 2019; Briffa et al., 2011).

The growth of new high technologies, such as artificial intelligence (AI), machine learning, and deep learning has changed the way medical doctors diagnose ACS in patients who cannot be diagnosed with inspection. Numerous studies have been carried out to facilitate the process of diagnosis by using machine learning techniques that reduce the time and resources required. Machine learning-based prediction shows the comparison of results between logistic regression (LR), gradient boosting machine (GBM), and artificial neural network (ANN) using electrocardiogram (ECG) data as a feature (Al-Zaiti et al., 2020). The diagnosis of ACS by using a support vector machine (SVM) with multiple features, such as age, gender, and cardiac enzymes of a patient, has been reported (Berikol, Yildiz & Özcan, 2016). Very recently, the diagnosis of myocardial infarction by using machine learning-based the myocardial-ischaemic-injury-index algorithm for a subset of ACS patients undergoing troponin measurement was reported (Doudesis et al., 2022). While all these studies have provided a potential prediction on ACS, only one of them has provided a classification model that can estimate the individualized likelihood of subgroups of myocardial infarction for patients with suspected ACS based on cardiac troponin concentrations. The main contribution of our research is the capability to predict ACS with the exact probability of recurrence risk for an individual patient based on patients’ medical records and to identify the significant features which have a high effect on ACS recurrence.

## Materials & Methods

### Experiment flow

**Figure 1:**
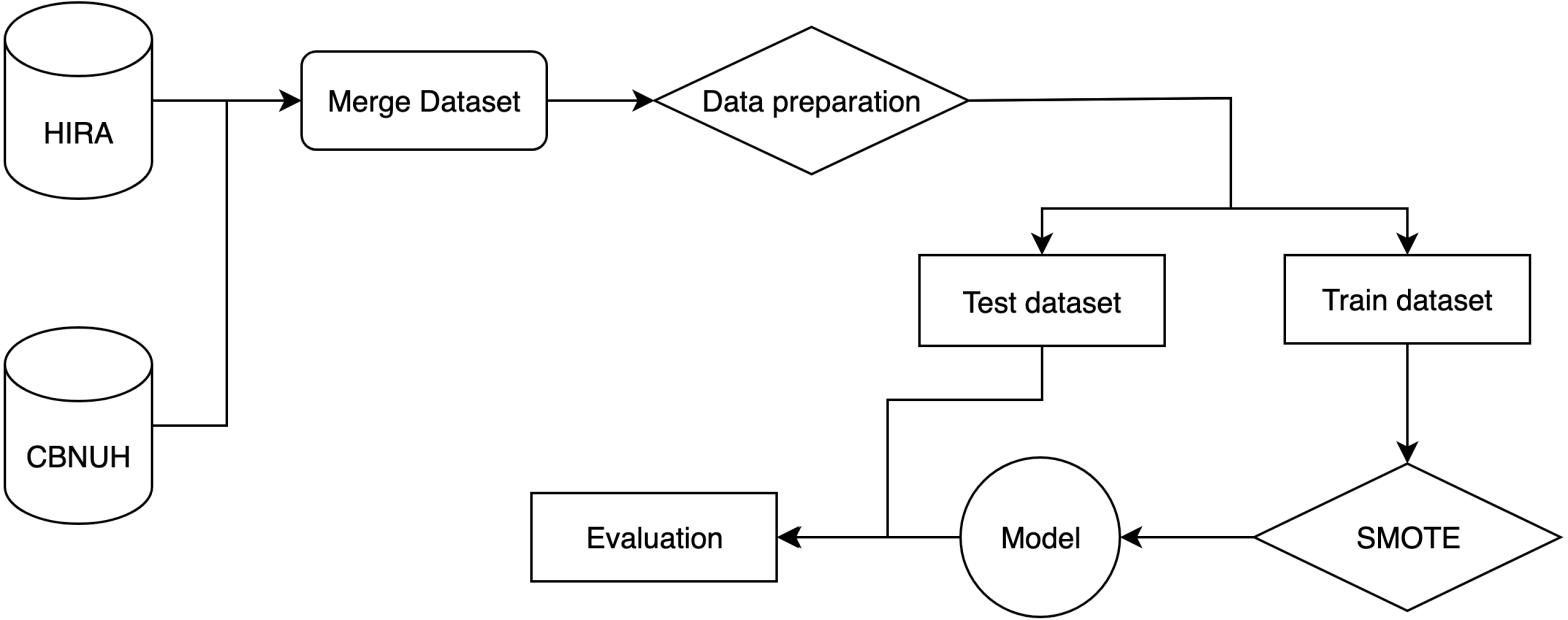
An overall end-to-end flow of the experiment.

The flow of the experiment is shown in Fig. 1. It starts by merging two datasets from two different data sources, HIRA and CBNUH, into a single dataset, which was followed by the data preprocessing step to handle missing data and invalid data format by removing invalid data and replacing with a unique number to differentiate it from valid data. Then, we split the dataset into two parts, which are the training dataset and test dataset for 70% and 30%, respectively. We applied the SMOTE algorithm to handle the problem of imbalance in the training dataset. Then, the training dataset was used to build the classification model, while the test dataset was used to evaluate the model performance.

### Patient datasets

This research article was mainly conducted with two different data sources: data from Korea Health Insurance Review and Assessment Service (HIRA) and patient data from Chungbuk National University Hospital (CBNUH), South Korea. A total of 947,788 records of 6,535 patients were used for the study including HIRA data of 945,659 records of 6,531 individuals who had been diagnosed with acute coronary syndrome (ACS) and CBNUH data of 2,129 records of four individuals who had been diagnosed with ACS and were outpatients for cardiac rehabilitation after percutaneous coronary intervention (PCI) at CBNUH (Table 1). Recurrence of ACS was defined as more than two hospitalizations for ACS within 12 months. This research was approved by the Institutional Review Board (IRB) at Chungbuk National University Hospital (IRB # CBNUH 2019-04-011-001) to fully use the provided dataset of CBNUH patients. All participants at CBNUH provided written consent prior to study participation. The IRB at CBNUH approved this study. All patient records of HIRA were de-identified and analyzed retrospectively, and as such, no informed consent was required. These two datasets were merged and the common predictors from both datasets were selected. The datasets are not balanced (skewed class) between the recurrent and control group of patients which was solved by applying the oversampling technique.

**Table 1:** Description of datasets used in the experiment.

**Data source**	**Recurrent patient**	**Control patient**	**Total patient**
CBNUH	0	4	4
HIRA	703	5828	6531
Total	703	5832	6535

### Prediction outcomes

The classification model that we built in this research paper produces two different outcomes. The first outcome is a binary result which represents the recurrence of ACS: “1” if the patient is predicted to have a recurrence and “0” if not. The second outcome is the probability of the patient’s risk which indicates how likely the patient will have a recurrence of ACS.

### Predictors

Variables for predictors were selected from data in both HIRA and CBNUH datasets. Predictor variables include demographics and medical history of an individual patient such as age, gender, procedure codes indicating procedures that each patient received, procedure reason for which patient was treated, prescription drug codes for which medication was prescribed, and condition codes indicating a patient’s diagnosis. These predictor variables are described in the following table (Table 2). The predictor variables were categorical variables except age, which was used as continuous variable.

The codes used in HIRA and CBNUH were manually converted into matching SNOMED-CT codes. Procedure codes include 41339005 for coronary angioplasty, 11101003 for percutaneous transluminal coronary angioplasty, 36969009 for placement of a stent in a coronary artery, and 415070008 for percutaneous coronary intervention. Procedure reason codes treated include 57054005 for acute transmural myocardial infarction of an unspecified site, 54329005 for acute transmural myocardial infarction of the anterior wall, 58612006 for acute transmural myocardial infarction of other sites (which was combined with 57054005 for acute transmural myocardial infarction of an unspecified site because the zero frequency of no recurrence for acute transmural myocardial infarction of an unspecified site did not allow us to estimate odds ratio), 73795002 for acute transmural myocardial infarction of the inferior wall, and 70422006 for acute subendocardial myocardial infarction. Prescription drug codes include 309362 for clopidogrel, 540788 for candesartan, 597977 for atorvastatin, 859747 for rosuvastatin calcium, and 246461 for aspirin. Condition codes include 57054005 for acute transmural myocardial infarction of an unspecified site, 371807002 for other forms of angina pectoris, 194828000 for unspecified angina pectoris, 54329005 for acute transmural myocardial infarction of the anterior wall, 73795002 for acute transmural myocardial infarction of the inferior wall, and 70422006 for acute subendocardial myocardial infarction.

**Table 2:** Description of training model’s predictor variables.

**Variable name**	**Explanation**
Age	Patient’s age
Gender	Patient’s gender
PRO_CODE	Procedure code (Ex: Percutaneous coronary intervention, etc.)
PRO_REASON_CODE	Procedure reason code for which patient was treated (Ex: Angina pectoris, etc.)
MED_CODE	Prescription drug code (Ex: Clopidogrel, etc.)
CON_CODE	Condition code in which patient’s condition was diagnosed (Ex: Acute myocardial infarction, etc.)

### SMOTE

Our dataset has more controls than recurrent patients, as shown in Table 1. To address the imbalanced nature of the dataset, the Synthetic Minority Oversampling Technique (SMOTE) was applied. The SMOTE algorithm was believed to solve the over-sampling problem to resample and rebalance the original dataset (Chawla et al., 2002). The new samples are created by interpolation between several minority class instances that are within a defined neighbourhood (Fernández et al., 2018). First, it randomly selects multiple nearest data points from the minority class before connecting all those data points together to create a line between them. The new sample is generated by picking random data points which are located on the connected lines (Chawla et al., 2002). In this study, the parameters of the SMOTE algorithm were set after several trials to find out the best setting that improved the model performance the most. We found out that setting ‘sampling_strategy’ = ‘not majority’, and ‘k_neighbors’ = 7 are the best parameter settings for our dataset by comparing the model performance with the rest of the setting trials.

### Logistic regression and model comparison

We performed machine learning techniques on the predictors to classify and predict the risk probability of patients. The dataset was pre-processed and fit into the logistic regression (LR) model: a categorical classification technique which generates the coefficient for each predictor. Odds ratios of each predictor were estimated with 95% confidence intervals and *P* values. LR was used for mapping qualitative or quantitative input features to a target variable, such as medical, financial, biological, or sociological data, whose prediction is attempted. If the labels are known, this is known as supervised learning in machine learning terminology (Kirasich, Smith & Sadler, 2018). The coefficient in LR represents the change of the dependent variable for one unit of change in the predictor variables while holding other predictors in the model constant (Jaccard, Lewis-Beck & Jaccard, 2001). Model performance was compared with multiple classification algorithms including naive Bayes, random forests, decision tree, support vector machines, and K-nearest neighbors.

### Experiment environment and libraries

The experiment was conducted using libraries and modules built using the Python programming language (v 3.9.10). Some of those important libraries are Statsmodels (v 0.13.2), SKLearn (v 1.0.2), Matplotlib (v 3.5.1), Pandas (v 1.3.5), and Numpy (v 1.23.0). During the experiment, a machine running on the Windows 10 operating system with 32GB of memory was used as the training machine for this experiment.

## Results

### Model performance

The performance of a model can be evaluated by various criteria. In this study, we chose the accuracy, precision, recall, F1-score, and area under the curve (AUC) as the evaluation criteria. As shown in Table 3, the classification model can predict with decent evaluation criteria. The accuracy is the ratio of the number of observations that were correctly predicted to the total number of observations. The precision represents the ratio of correctly predicted positive observations to the total predicted positive observations. The recall is the ratio of correctly predicted positive observations to all observations in the actual class. The F-1 score represents the harmonic mean of recall and precision. The above model can also indicate the probability of a recurrence of ACS for an individual patient (Table 4). The probability can give a clue on how likely a patient will have ACS recurrence in the future, which allows doctors and patients to estimate the patient’s risk more accurately.

### Performance comparison

**Figure 2:**
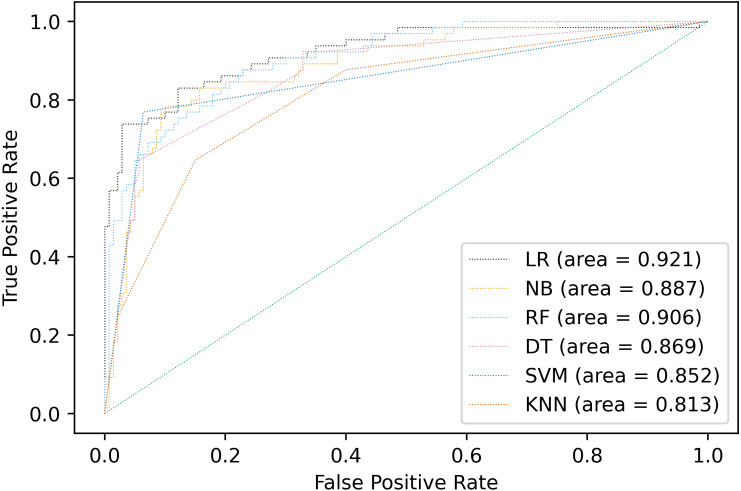
Comparison of AUC values of multiple models.

Multiple classification algorithms were compared including logistic regression, naive Bayes, random forests, decision tree, support vector machines, and K-nearest neighbors. The same dataset was used for all the algorithms, but the prediction results of the compared algorithms were shown by using accuracy, precision, recall, F1-score and AUC different depending on the model in Fig. 2 and Table 5. The best performance was observed in LR algorithm, which showed the highest prediction values in almost every evaluation criterion. Besides being the most outperformance, LR from Statsmodels is the preferable choice for disease classification because it provides easy access to all statistical metrics, including OR, which is easier for clinicians and medical staff to understand. LR would be the preferable choice among other algorithms because OR would be easily interpreted in the medical context.

**Table 3:** Logistic regression model performance using evaluation metrics.

**Accuracy**	**Precision**	**Recall**	**F1-Score**	**AUC**
0.893	0.894	0.851	0.869	0.921

**Table 4:** Binary and probability results of model prediction of 10 exemplary individual patients.

**Patient no.**	**Binary prediction**	**Probability prediction**
patient_1	0	0.263
patient_2	0	0.451
patient_3	1	0.859
patient_4	1	0.972
patient_5	0	0.358
patient_6	0	0.281
patient_7	0	0.472
patient_8	1	0.862
patient_9	0	0.359
patient_10	0	0.412

**Table 5:** Comparison of classification performance across multiple algorithms.

**Model type**	**Accuracy**	**Precision**	**Recall**	**F1-Score**	**AUC**
Logistic Regression	0.893	0.894	0.851	0.869	0.921
Naive Bayes	0.859	0.858	0.835	0.836	0.887
Random Forests	0.815	0.825	0.807	0.794	0.906
Decision Tree	0.810	0.812	0.787	0.783	0.869
Support Vector Machines	0.883	0.881	0.852	0.861	0.852
K-Nearest Neighbors	0.785	0.784	0.748	0.750	0.813

### Importance of predictors

In addition to building a model to classify patients with ACS, this study also identifies and ranks the variables based on their individual importance. As shown in Table 6, each variable has its OR, which represents how much the variable affects the classification model, and a *P*-value, which can be used to indicate the significance of the relationship between the feature and target variable. The table shows five statistically significant features that increase the risk of ACS recurrence including age, acute transmural myocardial infarction of an unspecified site and other sites over acute transmural myocardial infarction of the inferior wall (reference) of procedure reason, and acute transmural myocardial infarction of an unspecified site and unspecified angina pectoris over acute transmural myocardial infarction of the inferior wall (reference) of condition. The result also indicates five statistically significant features that reduce the risk of ACS recurrence including acute transmural myocardial infarction of the anterior wall and acute subendocardial myocardial infarction over acute transmural myocardial infarction of the inferior wall (reference) of procedure reason, and acute subendocardial myocardial infarction and acute transmural myocardial infarction of the anterior wall over acute transmural myocardial infarction of the inferior wall (reference) of condition.

**Table 6:** Statistical result of classification model using Logistic Regression Classification.

**Variable**	**Coefficient**	*P*-value	**Odds ratio**	**CI 95% (2.5%)**	**CI 95% (97.5%)**
**Age**	0.003	<0.001	1.003	1.002	1.004
**Gender**					
Male	Reference				
Female	−0.017	0.088	0.983	0.963	1.003
**Procedure**					
Percutaneous coronary intervention	Reference				
Percutaneous transluminal coronary angioplasty	0.001	0.999	1.001	0.370	2.711
Placement of a stent in a coronary artery	−0.007	0.962	0.993	0.738	1.336
Coronary angioplasty	−0.004	0.991	0.996	0.543	1.828
**Procedure reason**					
Acute transmural myocardial infarction of the inferior wall	Reference				
Acute transmural myocardial infarction of an unspecified site and other sites	4.584	<0.001	97.908	94.242	101.715
Acute transmural myocardial infarction of the anterior wall	−0.112	<0.001	0.894	0.864	0.925
Acute subendocardial myocardial infarction	−0.775	<0.001	0.461	0.448	0.474
**Prescription drug**					
Aspirin	Reference				
Clopidogrel	0.038	0.445	1.038	0.943	1.143
Candesartan	0.021	0.805	1.021	0.867	1.202
Atorvastatin	0.062	0.436	1.063	0.911	1.242
Rosuvastatin calcium	0.053	0.417	1.055	0.928	1.199
**Condition**					
Acute transmural myocardial infarction of the inferior wall	Reference				
Unspecified angina pectoris	0.224	<0.001	1.251	1.130	1.386
Other forms of angina pectoris	−0.013	0.866	0.987	0.847	1.150
Acute transmural myocardial infarction of an unspecified site	4.064	<0.001	58.215	50.479	67.136
Acute subendocardial myocardial infarction	−2.451	<0.001	0.086	0.077	0.096
Acute transmural myocardial infarction of the anterior wall	−0.902	<0.001	0.406	0.360	0.457

The ORs are critical evaluation criteria for a classification model. The OR represents the odds that an outcome will occur given a particular exposure, compared to the odds of the outcome occurring in the absence of that exposure (Szumilas, 2010). Based on the OR values, acute transmural myocardial infarction of an unspecified site and other sites as a procedure reason code has the highest odds ratio of 97.908 for ACS recurrence compared to the reference and the same acute transmural myocardial infarction of an unspecified site as a condition code also has the highest odds ratio of 58.215 for ACS recurrence compared to the reference, which indicates that when patient’s reason for hospital visit (procedure reason code) is acute transmural myocardial infarction of an unspecified site and other sites or when patient’s condition (condition code) is diagnosed as acute transmural myocardial infarction of an unspecified site, the patient’s risk for ACS recurrence increases tremendously.

## Discussion

ACS is a major public health concern all over the globe, which causes 1.8 million deaths per year (Padilla, Martín-Asenjo & Bueno, 2017). Cardiac events as well as these numerous deaths have a serious impact on the world economy which is a result of hospitalization expenses, such as coronary intervention, drugs, and/or surgery. An accurate diagnosis and timely proper treatment can save patients from losing their lives. It would be incredibly helpful if a doctor could give a prediction of ACS to a patient using the medical history and records of those patients. Artificial Intelligence (AI), machine learning, and deep learning algorithms are trending technologies in identifying the risk factors and classifying the recurrence of a patient.

Several studies have shown significant results on ACS classification. Classification of ACS with an electrocardiogram (ECG) provides important information about the presence, extent, and severity of myocardial ischemia (Birnbaum et al., 2014). Four different algorithms, support vector machine (SVM), artificial neural network (ANN), naïve bayes, and logistic regression, have been reported to find the most accurate model (Berikol, Yildiz & Özcan, 2016). The results show that SVM gave the highest accuracy among the four algorithms which showed a 99.13% accuracy for 228 patients. An AI model identified ACS by differentiating patients with myocardial infarctions from those with unstable angina (Salari et al., 2013). Similar to the previous studies, our study can also classify the status of ACS recurrence in patients with accuracies of 83.5% to 88.1% except 55.2% of Native Bayes. Among the variables of our study, acute transmural myocardial infarction of an unspecified site as a procedure reason and a condition contributed most significantly to ACS recurrence. acute transmural myocardial infarction of an unspecified site. Acute transmural myocardial infarction of an unspecified site has been reported as one of the patient’s conditions that increases mortality of patients with acute myocardial infarction as well as female and age (Baek et al., 2019).

Furthermore, our study also provides the probability of the ACS recurrence of each patient. This probability indicates the likelihood of ACS recurrence for a patient, which helps doctors and patients to more accurately estimate a patient’s risk. By providing the probability of ACS recurrence for each patient, the patient may be more motivated to participate in cardiac rehabilitation at the hospital or home (Kraal et al., 2017; Rah et al., 2020). For ACS patients, cardiac rehabilitation of 6 to 12 weeks after discharge has been reported to reduce the recurrence of ACS and mortality as secondary prevention (Kim, 2016; Redfern et al., 2007; Wenger, 2008). However, current participation rates in cardiac rehabilitation are below 40% due to various reasons, including difficult transportation and lack of need (Evenson & Fleury, 2000; Cortés & Arthur, 2006; Grace et al., 2005). Previous studies classified ACS recurrence risk on population level; however, they did not provide the probability of individual patients (Birnbaum et al., 2014; Salari et al., 2013). What makes our study unique is that we applied a currently available machine learning algorithm using logistic regression to estimate ACS recurrence risk of individual patients and provided an exact probability to each patient as a personalized ACS recurrence risk. By providing this probability to each patient as a personalized ACS recurrence risk in a clinical context, the patient may become more eager and motivated to reduce their own ACS recurrence risk.

Our study had several limitations. First, we chose variables to predict ACS recurrence risk which have data in both HIRA and CBNUH datasets. Some data points that do not exist in both datasets were not used, including a patient’s lifelog data, that may have effects on the recurrence risk of ACS as well. Second, a novel algorithm to estimate ACS recurrence risk of individual patients was not developed in our study; however, we applied already available technology to make it more meaningful to each ACS patient. Third, the uncommonly high odds ratios in our results may come from imbalanced distributions of the variables in our dataset. Because sparse or small sample size is considered as an important limitation in estimating ORs, our results should be interpreted cautiously.

## Conclusions

We used two real datasets, HIRA and CBNUH, to build a machine learning model which can classify ACS with high evaluation criteria. The model provides the probability of ACS recurrence of a patient as a personalized ACS recurrence risk, which can motivate ACS patients to reduce their own ACS recurrence risk such as cardiac rehabilitation. The model also shows that acute transmural myocardial infarction of an unspecified site and other sites, one of the conditions of acute myocardial infarction contributed most significantly to ACS recurrence with an odds ratio of 97.908 as a procedure reason code and with an odds ratio of 58.215 as a condition code.

## Additional Information and Declarations

### Data Availability

The following information was supplied regarding data availability: The data is available at Gitlab: https://gitlab.com/kongvungsovanreach/acs-recurrence-risk-prediction.
